# Matrix Development in Self-Assembly of Articular Cartilage

**DOI:** 10.1371/journal.pone.0002795

**Published:** 2008-07-30

**Authors:** Gidon Ofek, Christopher M. Revell, Jerry C. Hu, David D. Allison, K. Jane Grande-Allen, Kyriacos A. Athanasiou

**Affiliations:** Department of Bioengineering, Rice University, Houston, Texas, United States of America; Massachusetts Institute of Technology, United States of America

## Abstract

**Background:**

Articular cartilage is a highly functional tissue which covers the ends of long bones and serves to ensure proper joint movement. A tissue engineering approach that recapitulates the developmental characteristics of articular cartilage can be used to examine the maturation and degeneration of cartilage and produce fully functional neotissue replacements for diseased tissue.

**Methodology/Principal Findings:**

This study examined the development of articular cartilage neotissue within a self-assembling process in two phases. In the first phase, articular cartilage constructs were examined at 1, 4, 7, 10, 14, 28, 42, and 56 days immunohistochemically, histologically, and through biochemical analysis for total collagen and glycosaminoglycan (GAG) content. Based on statistical changes in GAG and collagen levels, four time points from the first phase (7, 14, 28, and 56 days) were chosen to carry into the second phase, where the constructs were studied in terms of their mechanical characteristics, relative amounts of collagen types II and VI, and specific GAG types (chondroitin 4-sulfate, chondroitin 6-sulfate, dermatan sulfate, and hyaluronan). Collagen type VI was present in initial abundance and then localized to a pericellular distribution at 4 wks. N-cadherin activity also spiked at early stages of neotissue development, suggesting that self-assembly is mediated through a minimization of free energy. The percentage of collagen type II to total collagen significantly increased over time, while the proportion of collagen type VI to total collagen decreased between 1 and 2 wks. The chondroitin 6- to 4- sulfate ratio decreased steadily during construct maturation. In addition, the compressive properties reached a plateau and tensile characteristics peaked at 4 wks.

**Conclusions/Significance:**

The indices of cartilage formation examined in this study suggest that tissue maturation in self-assembled articular cartilage mirrors known developmental processes for native tissue. In terms of tissue engineering, it is suggested that exogenous stimulation may be necessary after 4 wks to further augment the functionality of developing constructs.

## Introduction

Articular cartilage is a specialized type of hyaline cartilage, providing a nearly frictionless surface along diarthrodial joints and acting to resist and distribute compressive forces. Its tensile and compressive properties are attributed to the presence of fibrillar collagens and negatively charged glycosaminoglycans (GAGs), respectively, in the tissue's extracellular matrix (ECM). Articular cartilage lacks the ability to repair itself under conditions of wear and tear or traumatic injury [1], leading to osteoarthritis (OA) which afflicts millions of Americans and significantly affects the economy [2]. In recent years, tissue engineering has become a promising option toward the treatment of OA, allowing researchers to produce functional replacements for diseased cartilage.

Toward this end, a self-assembling process has been developed in our laboratory to yield cartilage tissue constructs of clinically relevant dimensions and compressive mechanical properties approaching those of native tissue [3]–[5]. Most notably, this approach does not involve the use of a scaffold, thereby bypassing the typical scaffold-related concerns of biodegradability, stress-shielding, and hindrance of cell-to-cell communication. While previous studies have focused on the end functionality of tissue constructs, an understanding of the development of neotissue within the self-assembling process remains incomplete. Studying the maturation of these cartilage constructs will yield valuable information regarding the developing biophysical environment of chondrocytes and elucidate intervention windows for biochemical or biomechanical stimulation.

An understanding of native articular cartilage development is of particular importance, as it will provide essential benchmarks for tissue growth *in vitro*. The various ECM components, and their associated arrangement, can be used as indicators for the effectiveness of a myriad of tissue engineering approaches, including the self-assembling process, to recapitulate the different stages of the developmental process of articular cartilage and produce functional tissue constructs [6]. Native articular cartilage is known to arise during an intricate process of joint development, first involving the formation of an interzone region through mesenchymal condensation at the future joint site, which then separates in a perichondrium-like layer and an intermediate layer consisting of softer tissues. Nascent cartilage begins to form with the chondrogenesis of mesenchymal progenitor cells located at the perichondrium regions and continues to develop until the formation of growth plates, which become the primary source of self-renewing, proliferating chondrocytes [7]. Like many musculoskeletal tissues, the biochemical composition of articular cartilage undergoes substantial changes during its development, reflected particularly in modulating levels and spatial organization of specific collagen and GAG types [8], [9]. In particular, it has been observed that collagen type VI plays an essential role in cartilage development and the maintenance of the cellular microenvironment [10], [11]. Variations in chondroitin sulfation patterns may be further indicative of matrix remodeling [12], [13], and thus affect the binding and activity of growth factors or cytokines within the tissue matrix. Correlating with these biochemical alterations, the compressive and tensile mechanical properties of articular cartilage also change during fetal and adolescent tissue maturation [14]–[17].

The purpose of this study was to examine the development of tissue within a self-assembling process for articular cartilage. Specific emphasis was placed on the relative levels of collagen types II and VI, and chondroitin 4-sulfate (CS-4) and chondroitin 6-sulfate (CS-6), and mechanisms of cellular aggregation during the early stages of neotissue development. We hypothesized that the relative levels of specific collagens (types II and VI) and GAGs (CS-4 and CS-6) would follow known developmental trends for native articular cartilage. We further examined the temporal-spatial relationship of collagen types II and VI to identify the progression of a pericellular matrix (PCM) within the tissue. Additionally, this study investigated the compressive and tensile mechanical characteristics of the constructs, and their relationships to changing biochemical properties.

## Materials and Methods

This study was performed in two phases to examine tissue development within the self-assembling process for articular cartilage. In the first phase, tissue constructs were assessed histologically and quantitatively for their total collagen and GAG content after 1, 4, 7, 10, 14, 28, 42, and 56 days of development. In the second phase, four time points from the first phase (7, 14, 28, and 56 days) were chosen to examine maturing structure – function relationships in the tissue. To this end, tensile and compressive mechanical properties of the tissue constructs were related to collagen and GAG levels. The specific types of collagen (types II and VI) and GAGs (CS-4, CS-6, dermatan sulfate, and hyaluronan) were further investigated to identify key matrix developmental trends. The seeding and culture techniques were the same for both phases of this study.

### Cell isolation and seeding

Articular chondrocytes were isolated from the distal femur of 1 wk old male calves (Research 87 Inc., Boston, MA) less than 36 h post slaughter, via an overnight digestion in 0.2% collagenase type II (Worthington, Lakewood, NJ), as described previously [3]. To reduce variability among animals, a mixture of cells were pooled together from six and seven animals for the first and second phases of the study, respectively, to yield a representative mixture of chondrocytes. The pooled cells were counted on a hemocytometer, and their viability was assessed using a trypan blue exclusion test. Each femur yielded approximately 150 million chondrocytes and a viability of >99% was determined for all specimens. Chondrocytes were frozen in culture medium supplemented with 20% FBS and 10% DMSO at −80°C for 2 days before use. After thawing, viability remained >80%. Chondrocytes were centrifuged at 1.2×10^3^ rpm for 7 min and resuspended at a density of 5.5×10^7^ cells/ml in a chemically-defined medium, consisting of DMEM with 4.5 g/L-glucose and L-glutamine, 100 nM dexamethasone, 1% fungizone, 1% penicillin/streptomycin (Biowhittaker/Cambrex, Walkersville, MD), 1% ITS+ (BD Biosciences, Bedford, MA), 50 mg/mL ascorbate-2-phosphate, 40 mg/mL L- proline, and 100 mg/mL sodium pyruvate (Fisher Scientific, Pittsburgh, PA). Articular cartilage constructs were seeded by adding 100 µl of this cell suspension to custom-made 5 mm agarose coated wells (described below). Time t = 0 was defined as this point of initial construct seeding. Cells were given 3 h to coalesce and then a remaining 400 µl of medium was added to each well. Medium was subsequently changed every 24 h. Based upon prior results in our laboratory demonstrating enhanced tissue mechanical properties [4], self-assembled constructs were removed from confinement in the agarose well at 2 wks and transferred into 10-mm diameter wells coated with 2% agarose, where they remained up to 8 wks.

### Preparation of agarose wells for chondrocyte seeding

Agarose coated wells were constructed as described previously [5], [18]. Briefly, a negative polysulfone mold consisting of 5-mm diameter×10-mm long cylindrical prongs was constructed to fit into 6 wells of a 48-well plate (Costar, Corning, NY). Individual wells were constructed by pressing the negative mold into 1 ml of sterilized, molten 2% molecular biology grade agarose in phosphate-buffered saline (PBS) (Sigma, St. Louis, MO). The agarose was allowed to gel at room temperature for 1 h with the mold in place. To each agarose well, 500 µl of chemically-defined medium was added and changed twice to saturate the well by the time of cell seeding.

### Histology and immunohistochemistry

Samples from both phases were frozen in cryoembedding medium and sectioned at a thickness of 12 µm. Safranin-O and fast green staining were used to visualize the GAG distribution within the constructs [19]. Additional slides were processed for qualitative immunohistochemistry (IHC) examination of the presence and spatial arrangement of collagen type I, II, and VI, using a Biogenex i600 autostainer (San Ramon, CA). After fixation in 4°C acetone, the slides were washed with a solution of PBS containing tween, quenched of exogenous peroxidase activity with 1% hydrogen peroxide in methanol and blocked with serum (Vectastain ABC kit, Burlingame, CA). The slides were then incubated with either mouse anti-collagen type I antibody (Axell, Westbury, N.Y.) at a 1:750 dilution in PBS, rabbit anti- collagen type II antibody (Cederlane, Burlington, NC) at a 1:500 dilution in PBS, rabbit anti- collagen type VI antibody (US Biological, Swampscott, MA) at a 1:300 dilution in PBS, or rabbit anti-collagen type X antibody (Abcam Inc., Cambridge, MA) at a 1:300 dilution in PBS. The appropriate mouse or rabbit secondary antibody (Vectastain ABC kit) was applied, followed by the avidin-biotinylated enzyme complex (Vectastain ABC kit) and DAB reagent (Vector Labs). Slides were removed from the autostainer, counterstained with hematoxylin, dehydrated in graded ethanol, and mounted with a coverslip. Slides stained without the addition of a primary antibody served as negative controls. Native meniscal fibrocartilage and articular cartilage served as positive controls for collagen types I and II, respectively. The presence of N-cadherins was also examined on slides from Phase I and in a non-seeded cell suspension. Briefly, slides were fixed in 4% paraformaldehyde, blocked with 10% FBS, and incubated with a rabbit anti-N-cadherin primary antibody (US Biological, Swampscott, MA) at a 1:240 dilution in 1% BSA in PBS, followed by detection with a goat anti-rabbit secondary antibody (Alexaflour 546). Nuclei were observed using a Hoescht's stain. Porcine cardiac tissue served as a positive control for N-cadherin immunohistochemistry.

### Biochemical analysis

Approximately 3–4 mg pieces of each construct (n = 6 per time point) were frozen for biochemical analysis in both phases. Frozen tissue pieces were lyophilized and digested in pepsin (10 mg/ml) followed by pancreatic elastase (1 mg/ml) in acetic acid and Tris buffer solutions, respectively. Total DNA content was measured by Picogreen^®^ Cell Proliferation Assay Kit (Molecular Probes, Eugene, OR). Total sulfated GAG was then quantified using the Blyscan GAG assay kit (Biocolor, Newtownabbey, Northern Ireland), based on the 1,9-dimethylmethylene blue binding [20], [21]. Samples were further assayed for total collagen content via hydrolysis in 2N NaOH, followed by a chloramine-T hydroxyproline reaction, as described previously [22]. Specific levels of collagen type II were determined for Phase II samples using an Enzyme-Linked ImmunoSorbent Assay (ELISA) assay, developed by Chondrex, Inc. (Chondrex, Redmond, WA). Collagen type I was quantified via a sandwich ELISA using a monoclonal mouse anti-human capture antibody (USBiological) and polyclonal rabbit anti-human detection antibody (USBiological).

The relative levels of collagen type VI were examined through western blotting in Phase II. Based upon the results of the hydroxyproline assay, the equivalent amount of each tissue digest containing 20 µg of total collagen was precipitated for blotting. The samples, collagen type VI protein standard, and a prestained SDS-PAGE protein ladder (Bio-Rad) were loaded into a 10–20% Ready Gel Tris-HCl Gel (Bio-Rad) and run at 50 mA for 30 min. The gels were then transferred onto blotting paper at 350 mA for 60 min. Blots were blocked in 2% BSA for 3 h at room temperature and then incubated with a rabbit anti-COL6 antibody (USBiological, Swampscott, MA) at a 1:1500 dilution. A rabbit secondary antibody (Vectastain ABC kit) was applied, followed by the avidin-biotinylated enzyme complex (Vectastain ABC kit) and DAB reagent (Vector Labs) to visualize protein bands. The integrated optical density of each band was determined using GelPro^®^ software (Media Cybernetics, Bethesda, MD).

Fluorophore-assisted carbohydrate electrophoresis (FACE) analysis was additionally performed on Phase II samples (n = 6) to quantitatively determine changes in CS-4, CS-6, glucose, dermatan sulfate, and hyaluronan during construct development, as previously described [13], [23], [24]. Separate tissue construct portions were frozen, lyophilized, and rehydrated in 100 mM ammonium acetate. Tissue pieces were digested in 10% (w/v) proteinase-K (EMD Pharmaceutical, Durham, NC) overnight at 60°C. Ammonium acetate (100 mM) was subsequently added to each sample, and followed by digestion with either chondroitinase AC II alone or together with chondroitinase ABC (Associates of Cape Cod, Falmouth, MA). The difference in band intensity from the two digests yielded dermatan sulfate content. Samples were fluorescently tagged with with 2-aminoacridone HCl (Molecular Probes, Eugene, Oreg., USA) and run on a carbohydrate electrophoresis gel at 400 mA for 45–60 min. Individual FACE gel bands were analyzed using GelPro^®^ software (Media Cybernetics, Bethesda, MD). Specific GAGs and total glucose were quantified via comparison to a fluorescently labeled maltotriose standard curve.

### Mechanical assessment

The compressive mechanical properties of Phase II samples (n = 6 per time point) were evaluated with an indentation apparatus [25]. A 3-mm punch was extracted from each construct, attached to the sample holder with cyanoacrylate glue, and submerged in PBS. The sample was positioned under the load shaft of the apparatus, such that the sample surface test point was perpendicular to the indenter tip. A tare load of 0.2 g (0.002 N) was applied using a 1-mm diameter rigid, flat-ended, porous indenter tip and samples were allowed to reach tare creep equilibrium. A step load of 0.7 g (0.007 N) was then applied and sample displacement was measured until equilibrium was reached. The intrinsic mechanical properties of the samples, aggregate modulus (H_A_), permeability (*k*), and Poisson's ratio (υ), were determined using the linear biphasic theory [26], [27].

Tensile tests were performed on Phase II samples (n = 6) with an electromechanical materials testing system (Instron Model 5565, Canton, MA) using a 50 N load cell. Samples were cut into a dog-bone shape with an approximate gauge length of 1.5-mm and glued onto paper tabs for gripping. The thickness and width of each sample were appropriately measured to calculate the cross sectional area for the applied forces. Samples were pulled at a constant strain rate of 0.01 s^−1^. Stress-strain curves were developed from the load-displacement curve and analyzed for the tissue's tensile Young's modulus (*E_Y_*) and ultimate tensile strength (UTS).

### Statistical analysis

A single factor ANOVA was used with a Tukey's post-hoc when warranted. Significance was defined as *p*<0.05 throughout the study.

## Results

Based upon statistical changes in total collagen/wet weight (WW) and GAG/WW levels within the constructs in Phase I, the following four time points were selected for further investigation in Phase II: Day 7, Day 14, Day 28, and Day 56.

### Gross appearance

**Figure 1:**
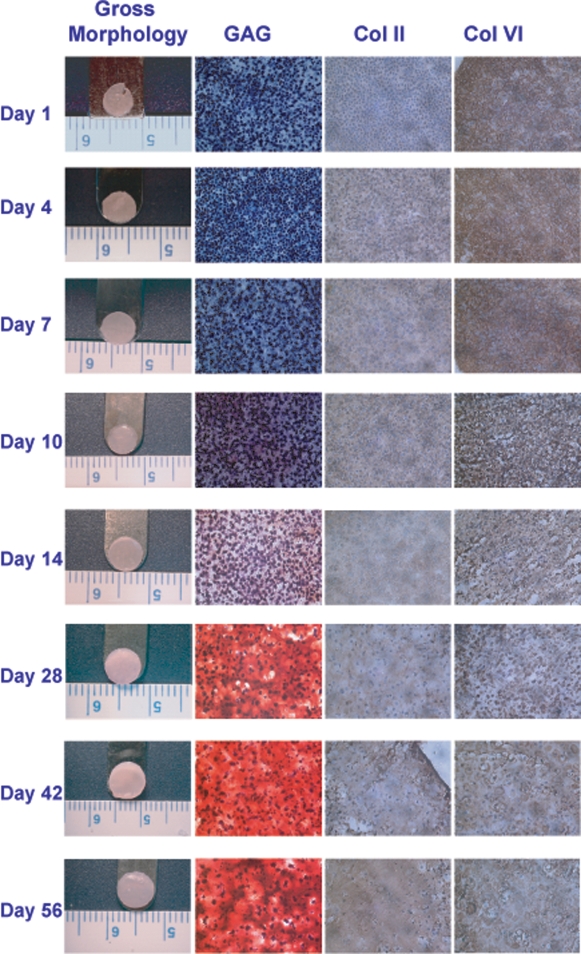
Gross morphology (column 1) and histological sections (columns 2–4) of self-assembled cartilage constructs. Original magnification, 40X. GAGs were initially concentrated pericellularly, but were observed throughout the tissue by 4 wks. Also at 4 wks, collagen type VI localized around the cells, while collagen type II appeared evenly distributed in the constructs.

The physical characteristics of self-assembled engineered articular cartilage constructs followed similar trends in development during both experimental phases (Table 1). At day 1, constructs appeared non-uniform and did not have smooth surfaces. Beginning at day 7, the surfaces of the construct began to glisten, suggesting an increased hydration and presence of GAGs (Fig. 1). This glistening appearance consistently became more evident over time. Finally, from 4 wks onward, the tissue constructs took on a very slight “bowl shaped” morphology.

**Table 1:** Physical characteristics of self-assembled articular cartilage constructs.

Phase I	Wet Weight (mg)	Thickness (mm)	Diameter (mm)
Day 1	4.2±2.8	0.4±0.1	5.0±0.2
Day 4	6.7±1.3	0.4±0.1	5.2±0.3
Day 7	7.0±1.3	0.4±0.1	5.5±0.2
Day 10	8.9±1.0	0.5±0.1	5.3±0.2
Day 14	10.1±1.1	0.5±0.1	5.3±0.2
Day 28	15.9±2.7	0.6±0.2	5.9±0.2
Day 42	25.7±3.7	0.6±0.2	6.3±0.1
Day 56	35.9±1.8	0.8±0.2	7.3±0.1
**Phase II**			
Week 1	5.6±0.4	0.3±0.1	5.0±0.2
Week 2	8.9±0.5	0.4±0.1	5.0±0.1
Week 4	18.6±0.8	0.6±0.1	5.9±0.1
Week 8	34.3±5.8	0.8±0.1	7.1±0.2

### Histology and immunohistochemistry

The spatial arrangement of GAGs and collagen type II and VI was modulated throughout the course of neotissue development (Fig. 1). Safranin-O staining for GAGs was observed in a pericellular distribution initially, with the staining intensity increasing over time throughout the construct. Collagen type II appeared evenly distributed within the ECM, with its staining intensity increasing uniformly over time. Interestingly, collagen type VI staining was observed in abundance initially throughout the tissue matrix and then could be seen localized around individual cells by 4 wks. Based on IHC, there was no collagen types I or X production at any time point, indicating no cellular dedifferentiation or hypertrophy in the constructs. Additionally, N-cadherin staining demonstrated a minimal presence of the protein in cell suspension. During the first day, however, the intensity of N-cadherin staining increased drastically and peaked at 4 days. Minimal N-cadherin staining could be observed onwards of 2 wks. Similar histology pictures could be seen for Phase II (not shown) as was observed for Phase I.

### Biochemical analysis

**Figure 2:**
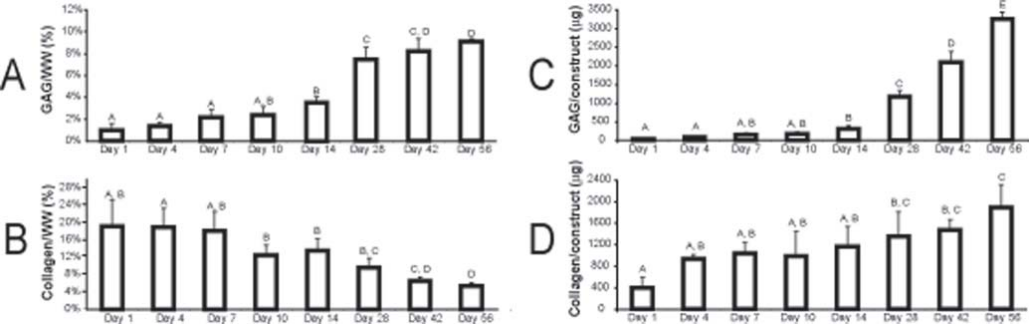
GAG and collagen content in constructs during development in Phase I. Significant differences in GAG/WW can be observed between 7 and 14 days, 14 and 28 days, and 28 and 56 days (A). Significant differences can be observed in collagen/WW between 7 and 14 days, and 28 and 56 days (B). Increasing trends were observed for both total GAG per construct (C) and total collagen per construct (D). Data presented as mean±standard deviations.

In the first phase, an increasing trend for GAG/WW was observed, with statistically significant increases occurring between days 7 and 14, days 14 and 28, and days 28 and 56. The GAG/WW values were 1.0±0.6%, 1.3±0.4%, 1.9±0.2%, 2.1±0.7%, 3.5±0.8%, 7.5±1.1%, 8.2±1.2%, and 9.1±0.3%, at days 1, 4, 7, 10, 14, 28, 42, and 56, respectively. Total GAG per construct also increased during tissue development, attaining a maximum value of 3.2±0.2 mg at 8 wks. In contrast to GAG/WW, a decreasing trend was found for total collagen/WW, with statistically significant changes between days 28 and 56. However, total collagen per construct displayed an increasing trend over time reaching a value of 1.9±0.3 mg at 8 wks. The collagen/WW values were 19.0±6.2%, 19.0±4.4%, 18.1±4.4%, 12.5±2.4%, 12.0±2.7%, 9.6±2.1%, 6.5±0.9%, and 5.3±0.9%, at days 1, 4, 7, 10, 14, 28, 42, and 56, respectively (Fig. 2). There were no statistical differences in construct cellularity during neotissue maturation.

**Figure 3:**
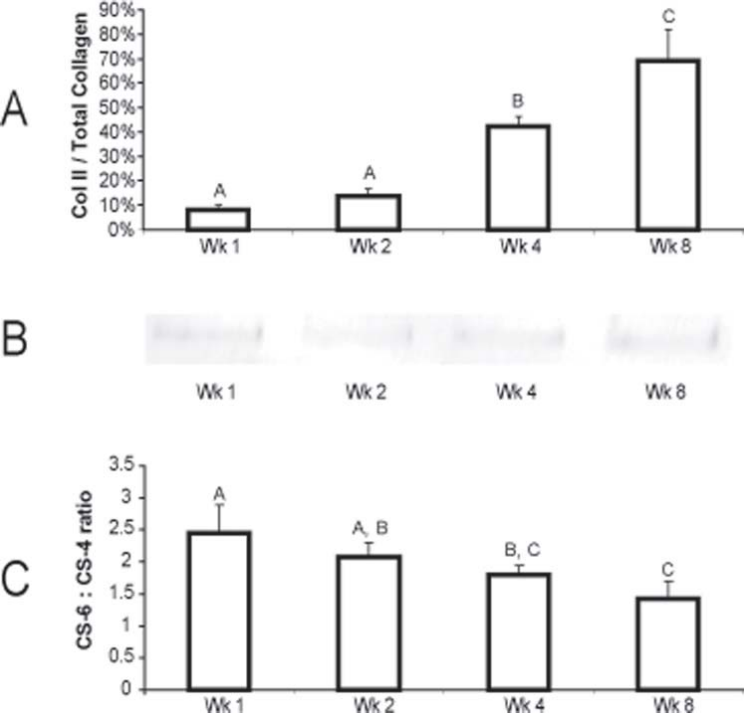
Three major biochemical changes can be observed in cartilage neotissue maturation with the self-assembling process, which mirror known developmental trends for articular cartilage. (A) The percentage of collagen type II to total collagen increased consistently over time, from 8.1% at 1 wk to 69.1% at 8 wks, with statistically significant increases between 2, 4, and 8 wks. (B) In a western blot for collagen type VI, normalized to total collagen content, it is observed that at the proportion of collagen type VI to total collagen decreased by approximately 40% between 1 and 2 wks and then increased slightly at 8 wks. Relative collagen type VI levels were determined by the integrated optical density of each band. (C) The ratio of CS-6 to CS-4 decreased steadily during construct maturation, from 2.4 at 1 wk to 1.4 at 8 wks. Data presented as mean±standard deviations.

Further investigation in Phase II into the specific levels of collagens type II and VI exposed differing trends. Through ELISA quantification, the levels of collagen type II/WW were 0.7±0.2%, 1.4±0.1%, 3.9±0.1%, and 4.9±0.7% for 1, 2, 4, and 8 wks, respectively, with statistical differences between 2 and 4 wks, and 4 and 8 wks. When normalized to total collagen, percentages of collagen type II to total collagen were 8.1±2.0%, 13.8±3.0%, 42.3±3.9%, and 69.1±12.9% for 1, 2, 4, and 8 wks, respectively (Fig. 3). Statistical changes were observed between 2 and 4 wks, and 4 and 8 wks. Through western blotting, the proportion of collagen type VI to total collagen also changed during development (Fig. 3). In the example blot, the integrated optical density of the band decreased by approximately 40% between 1 and 2 wks. No change was observed between 2 and 4 wks, and a 15% increase in band density occurred between 4 and 8 wks. Similar trends were observed in all samples tested. Collagen type I was not detectable at any time point with the ELISA.

Examination into the specific GAGs in Phase II samples revealed a decreasing ratio of CS-6 to CS-4 over time (Fig. 3). At 1, 2, 4, and 8 wks the relative levels of CS-6 to CS-4 was 2.5±0.4, 2.1±0.2, 1.8±0.1, and 1.4±0.3, respectively. Statistical differences were observed between 1 and 4 wks, 1 and 8 wks, and 2 and 8 wks. The total amount of chondroitin (0-, 4-, and 6-) sulfate was further quantified to display an increasing molar concentration over time. The total chondroitin sulfate values were 7.4±4.5 nmol/mg, 36.4±7.3 nmol/mg, 48.4±12.6 nmol/mg, and 64.5±5.3 nmol/mg for 1, 2, 4, and 8 wks, respectively, and statistical increases were recorded between 1 and 2 wks and 4 and 8 wks. Conversely, the total dermatan sulfate levels were 1.4±1.3 nmol/mg, 4.2±3.6 nmol/mg, 7.5±3.1 nmol/mg, and 5.3±1.7 nmol/mg for 1, 2, 4, and 8 wks, respectively, with a statistical difference observed only between 1 and 4 wks. Moreover, the amount of glucose in each sample was 0.2±0.2 nmol/mg, 0.6±0.2 nmol/mg, 3.1±1.1 nmol/mg, and 3.1±0.9 nmol/mg at 1, 2, 4, and 8 wks, respectively, with a statistical increase observed between 2 and 4 wks. Finally, hyaluronan was not observed in a measurable quantity at any time point.

**Figure 4:**
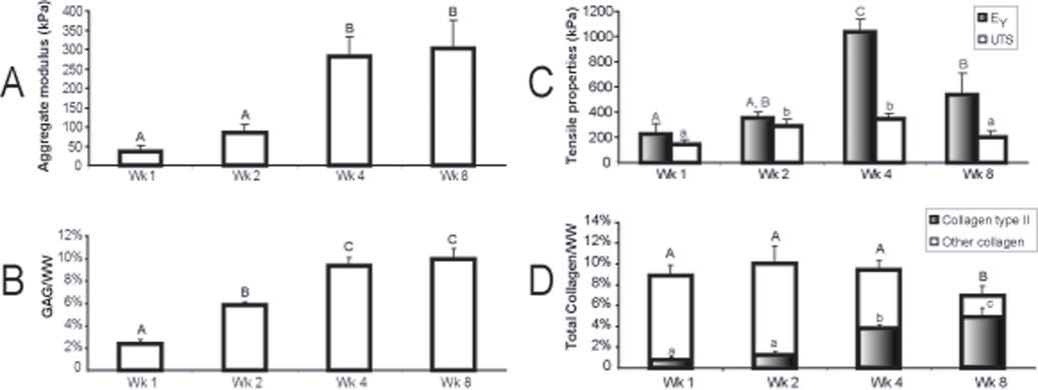
Biomechanical and biochemical properties of the constructs in Phase II. Large and significant increases in the aggregate moduli (A) were observed between 2 and 4 wks, corresponding to increases in GAG/WW (B). The Young's modulus and ultimate tensile strength (C) increased during the first 4 wks, coinciding with increases in the percentage of collagen type II to total collagen. Both tensile mechanical properties then decreased significantly at 8 wks, concurrent with a similarly significant decrease in total collagen/WW (D). Data presented as mean±standard deviations.

The second phase of this study also displayed similar values and trends for total GAG and collagen as the first phase (Fig. 4). The GAG/WW levels were 2.4±0.3%, 5.9±0.2%, 9.4±0.7%, and 10.0±0.1% for 1, 2, 4, and 8 wks, respectively. Statistical changes were observed for GAG/WW between 1 and 2 wks, and 2 and 4 wks. The collagen/WW levels were 8.9±1.0%, 10.0±1.6%, 9.4±0.9%, and 6.9±1.0% at 1, 2, 4, and 8 wks, respectively. A statistically significant drop in collagen/WW was observed between 4 and 8 wks.

### Mechanical assessment

The compressive and tensile properties of the constructs were assessed in Phase II (Fig. 4). The aggregate modulus of the constructs was 37.2±15.2 kPa, 85.7±19.4 kPa, 280.3±52.7 kPa, and 304.0±72.6 kPa, for 1, 2, 4, and 8 wks, respectively, with a statistically significant increase between 2 and 4 wks. A significant linear correlation (R^2^ = 0.96, p<0.05) was further observed between the mean aggregate modulus and GAG/WW of each group. The permeability of the constructs was 1.5±1.9 (×10^−15^) m^4^/N-s, 8.5±7.5 (×10^−15^) m^4^/N-s, 6.0±5.8 (×10^−15^) m^4^/N-s, and 11.0±4.5 (×10^−15^) m^4^/N-s at 1, 2, 4, and 8 wks, respectively. A significant difference in permeability was observed between 1 and 8 wks. The Poisson's ratio of the constructs also increased over time, with values of 0.1±0.1, 0.2±0.1, 0.3±0.1, 0.4±0.1 at 1, 2, 4, and 8 wks, respectively. Significant changes in Poisson's ratio were observed between 1 and 4 wks, 1 and 8 wks, and 2 and 8 wks.

The Young's modulus of the 4 wk constructs at 1035.4±96.4 kPa was statistically greater than any other time point. The ultimate tensile strength of the 2 and 4 wk constructs at 293.5±55.3 kPa and 342.7±52.3 kPa, respectively, were significantly different from the 1 and 8 wk groups at 143.4±38.7 kPa and 200.3±51.1 kPa, respectively. Univariate regression analysis revealed that total collagen/WW did not have a statistically significant relationship with either the ultimate tensile strength (R^2^ = 0.29, p = 0.46) or the Young's modulus (R^2^ = 0.001, p = 0.97) of each group. Similarly, collagen type II/WW was not statistically correlated to the Young's modulus (R^2^ = 0.49, p = 0.30) or the ultimate tensile strength (R^2^ = 0.10, p = 0.68) at each time point.

## Discussion

This study was designed to examine the maturation of neotissue within a self-assembling process to engineer articular cartilage over an 8 wk culture period. It is important to understand neotissue development, since this may elucidate potential intervention windows for biochemical or biomechanical stimulation, as well as provide an essential characterization of structure and function of tissue replacements. This study presents several notable findings pertaining to the self-assembling process. 1) We demonstrate that collagen type VI is present throughout the ECM at early stages of neotissue growth and then localizes to a PCM around 4 wks, thereby recapitulating a major characteristic of *in vivo* cartilage development [8]. 2) Our results suggest that the initial cellular aggregation and construct formation within the self-assembling process is mediated by cadherin-cadherin interactions. 3) The protein content for collagens type II and VI, and relative levels of CS-4 and CS-6, follow similar trends to that of native cartilage development during all 8 wks of culture. Similarly, tissue compressive and tensile mechanical properties mirrored developmental trends during the first 4 wks. 4) This study identifies 4 wks as a potential time point for exogenous stimulation to further augment the functional properties of the tissue constructs.

To our knowledge, this is the first study to demonstrate the localization of collagen type VI in a scaffold-less tissue engineering approach. Previous research has identified the protein in tissue engineered cartilage scaffolds [28], and chondrocyte-seeded agarose constructs [29], but not in a self-assembly cell-based approach which seems to capture the developmental nature of articular cartilage. Collagen type VI is the primary marker for the PCM [30], [31]. The PCM, together with its enclosed cell, is defined as the *chondron* and is considered to be the smallest metabolic and functional unit of articular cartilage [32]. In particular, it is known that the PCM surrounding the chondrocyte organizes and constructs collagen fibrils [32], regulates cellular osmolarity [33], [34], and modulates growth factor interactions with the enclosed cell [35]. The PCM also acts as a biomechanical buffer of applied stresses [36]–[39] and may play an important role in the mechanotransductive response of chondrocytes to exogenous stimulation within a tissue engineering approach. Therefore, future work may focus on investigating the three-dimensional structure of chondrons [40] in self-assembled cartilage constructs to further characterize the chondrocyte's developing microenvironment. In addition, chondrons can be isolated from cartilage constructs at various stages of development and directly tested to determine the mechanical properties of the newly-formed PCM [38], [41].

We propose that the early stages of tissue development within the self-assembling process are mediated by cadherin-cadherin interactions, as described by the differential adhesion hypothesis [42]. These interactions act to minimize the free energy of the population of cells by sorting cells with varying intracellular adhesiveness. In articular cartilage, it has been shown that N-cadherin activity spikes to its highest level during mesenchymal condensation [43] and subsequently decreases around the central condensation region, as the cells spread apart and begin to differentiate into chondrocytes. At later stages of joint development, N-cadherin staining is only evident in the perichondrium and not in mature cartilage tissue [44]. A similar trend is observed in the maturation of self-assembled engineered cartilage, suggesting that our process captures this integral component of native articular cartilage development. N-cadherins are minimally present in the initial cell suspension, and then are observed with an intense staining upon 1 day of seeding. Therefore, it can be inferred that N-cadherin activity plays a significant role in cellular aggregation during the self-assembling process. Indeed, this may have broad implications for future tissue engineering studies attempting to recapitulate the zonal architecture of various cartilaginous tissues through co-cultures of cells with varying cadherin levels.

Parallel trends in collagen content can be observed in the maturation of tissue within the self-assembling process and native articular cartilage development. In our study, collagen type II represented a small fraction (8%) of the total collagen at early stages of neotissue maturation. Over time, collagen type II levels significantly increased, reaching 69% of the total collagen at 8 wks. Similarly, collagen type II is not found in native tissue until after partuition, both in terms of mRNA expression or protein production [45]. In mature cartilage, however, collagen type II is observed throughout the ECM [8], [45] and composes approximately 85–90% of the total collagen [46]. It was further observed in this study that collagen type VI was present in high abundance initially, and then decreased in proportion to total collagen, as the protein localized around individual cells. An analogous trend has been observed in native articular cartilage for collagen type VI. The expression of collagen type VI spikes at chondrocyte differentiation [10] and is present throughout matrix development [8], [11]. This steep rise in collagen type VI expression in native tissue soon levels off at later stages of chondrogenesis, coinciding with an upregulation in collagen type II [10].

In terms of chondroitin sulfate, there are several similarities between the self-assembling process and native tissue maturation. The decreasing ratio of CS-6 to CS-4 (from 2.5 at 1 wk to 1.4 at 8 wks) observed in this study is mirrored in native cartilage tissue development. During embryogenesis, CS-6 can be found throughout the developing matrix, then from parturition onwards the staining intensity and relative abundance for the GAG increase uniformly [9]. Conversely, CS-4 is observed in trace amounts in the developing ECM [9], and then increases in content at a greater rate than CS-6 post parturition [47], [48]. However, it should be noted that species dependant sulfation patterns have been previously reported in developing tissues [49].

Similar trends can also be drawn between the maturing biomechanical properties of self-assembled articular cartilage and those of native tissue. Excitingly, this study observed analogous trends in compressive mechanical properties to reported results in the aggregate moduli of developing bovine articular cartilage. Our 2 wk (85±19 kPa), 4 (280±52 kPa), and 8 wk (304±72 kPa) values are right on par with the aggregate moduli of third trimester fetal (110±30 kPa), 1–3 wk old calf (270±20 kPa), and young adult bovine (310±30 kPa) articular cartilage explants, respectively, from the knee joint [16]. However, previous studies have shown that the stiffness of mature bovine cartilage, as measured by creep indentation, ranges from 472 kPa to 899 kPa [27], [50], suggesting that further increases in the compressive properties of self-assembled articular cartilage constructs may be necessary. Additional comparisons can be made between the changes in the emergent tensile properties of self-assembled cartilage constructs and the developing cartilage from the femoral condyle. Significant increases in the Young's moduli of our constructs between 2 and 4 wks and decreases between 4 and 8 wks mirrored the changes observed between third trimester fetal, 1–3 wk old calf, and young adult bovine articular cartilage [17]. These similarities in compressive and tensile properties are additionally noteworthy since our current study utilized immature bovine articular chondrocytes in the self-assembling process. Taken together with the observed trends in biochemical components, our results suggest that the self-assembling process embodies many of the known developmental characteristics for articular cartilage. Future studies, therefore, should look to identify key developmental signaling patterns, such as Wnt [51], [52] or bone morphogenetic protein expression [53], to confirm this tissue engineering approach as an appropriate model for *in vitro* cartilage development. Moreover, further research may examine matrix maturation during self-assembly of differentiated embryonic stem cells [54], which more closely resemble the phenotypic state of cells during the mesenchymal condensation phase of native articular cartilage development.

Statistically significant changes in the developing compressive and tensile mechanical properties of the neotissue were generally accompanied by concurrent alterations in GAG and collagen levels, respectively. Significant increases in GAG/WW between 1 wk (2.4%), 2 wk (5.9 %), and 4 wk (9.4%) coincided with a stiffening of the constructs (37.2 kPa to 85.7 kPa to 280.3 kPa). Moreover, a significant correlation was observed between GAG levels and aggregate moduli. Notably however, no change was observed in the aggregate modulus or GAG content after 4 wks, although the Poisson's ratio did continue to increase. This suggests that other ECM components, possibly cross-linking proteins collagen type IX or fibronectin, may influence the apparent compressibility of the constructs, and thus warrant further investigation. Similar relationships were observed between tensile properties and collagen content. Statistically greater ultimate tensile strength values at 2 and 4 wks (293.5 kPa and 342.7 kPa, respectively) over 1 and 8 wks (143.4 kPa and 200.3 kPa, respectively) followed similar trends in total collagen/WW. The statistically significant decrease in total collagen/WW between 4 and 8 wks also coincided with a significant drop in Young's moduli (1035.4 kPa to 537.9 kPa). The significant increase in Young's modulus between 2 and 4 wks, however, was not accompanied by changes in total collagen/WW. Therefore, it is possible that the observed tensile stiffening may be due to collagen organizational changes or increased cross-linking within the ECM. Additionally, this difference in tensile properties may be accounted for by an increased presence of collagen type II. Similarly, significant increases in Young's moduli and collagen type II/WW were observed between 2 and 4 wks, indicating that the specific type of collagen, not just total collagen content, present in the neotissue may dramatically influence tensile properties. Moreover, this result confirms the substantial role that collagen type II has on tensile characteristics [55], and therefore a preferred target for growth factor treatments to augment these properties [56].

Examining the structural and biochemical changes during tissue construct growth within the self-assembling process sheds light on potential intervention strategies employing exogenous stimulation. At 4 wks, the GAG content of our constructs (9.4% GAG/WW) far exceeds that of native tissue (∼5% GAG/WW) [46]. While it is important not to hinder the developing compressive properties, the application of small doses of chondroitinase-ABC [57] or other treatments may be helpful to reorganize the tissue matrix and bring GAG levels closer to those of native tissue. This is essential for later *in vivo* work where a mismatch between the osmolarity of the constructs and the surrounding host tissue is undesirable. Potential mechanical stimulation windows can also be identified. Our current finding of PCM formation after 4 wks of growth suggests that this is potentially an appropriate time point for compressive mechanical stimulation, since the PCM is known to appropriately facilitate the transmission of mechanical stresses onto the enclosed chondrocytes [36], [39], [58]. Moreover, our observation that the aggregate modulus reached a plateau at 4 wks, and the tensile Young's modulus decreased after 4 wks further warrants consideration that exogenous stimulation mechanisms may be necessary around that time point to further augment the functional characteristics of the neotissue. The statistical drop in total collagen/WW between 4 and 8 wks, coinciding with the decrease in tensile properties, is possibly due to increased activity of matrix metalloproteinases, and thus, exogenous tissue inhibitor of matrix metalloproteinases may be incorporated during this time period to prevent further breakdown of the collagen matrix. The slight increase in collagen type VI to total collagen between 4 and 8 wks may also signify greater matrix catabolism [59]. Moreover, the lack of hyaluronan production at any time point suggests that GAGs in our constructs are not being integrated into aggrecan aggregates, which is likely essential for a continued increase in compressive mechanical properties beyond 4 wks. Therefore, future studies should consider mechanisms to initiate hyaluronan production, such as hypoxia [60], growth factor stimulation [61], or inducible overexpression systems [62]. Finally, a significant increase in glucose retention was observed in the constructs between 2 and 4 wks, with no change thereafter. This drop in glucose consumption could be indicative of a concomitant decrease in chondrocyte metabolic activity during this period, which may necessitate dynamic mechanical stimulation [63] or greater concentrations of ascorbic acid added to the culture medium [64] at 4 wks.

**Figure 5:**
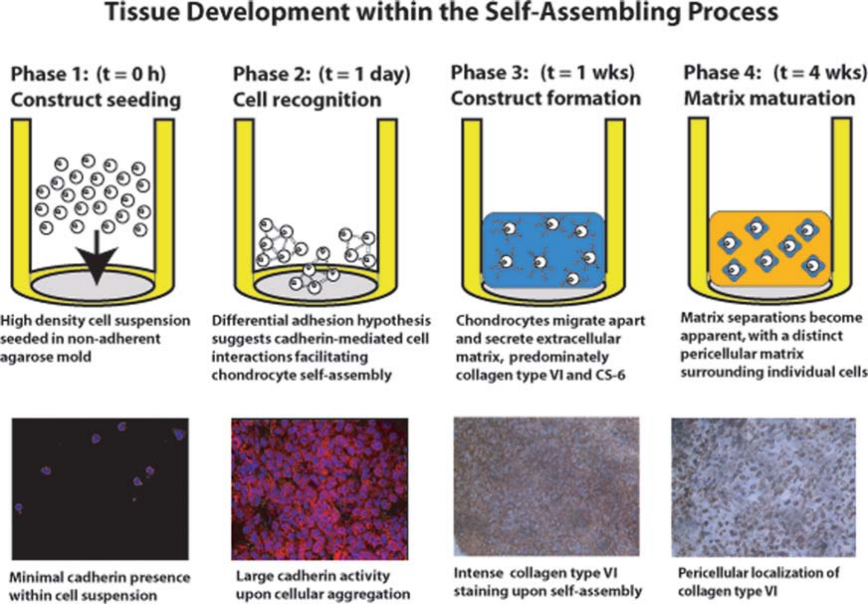
Tissue development within the self-assembling process can be described in four phases. In the first phase, a high density cell suspension is seeded in an agarose mold. In the second phase, chondrocytes begin to recognize each other and coalesce through a minimization of the free energy in the system, as described by the differential adhesion hypothesis. In the third phase, tissue constructs begin to form as the cells migrate apart and secrete predominately collagen type VI and CS-6 to compose its extracellular matrix. In the fourth phase, extracellular matrix separations become apparent, as indicated by a PCM surrounding individual chondrocytes. The initially abundant collagen type VI decreases in proportion to total collagen and localizes around individual cells, as the percentage of collagen type II to total collagen increases. The mature tissue matrix consists predominately of collagen type II and a slightly greater percentage of CS-6 than CS-4.

In conclusion, the development of neotissue within the self-assembling process can be described in four phases (Fig. 5). In the first phase, cells are seeded at a high density in a non-adherent agarose mold. Minimal N-cadherin activity is present at this stage, as the cells have not yet begun to recognize each other. In the second phase, N-cadherin activity increases, suggesting greater cell-cell interactions. According to the differential adhesion hypothesis, cells begin to coalesce at this stage to minimize the free energy within the biological system. In the third phase, cells begin to migrate apart and produce an ECM consisting of primarily collagen type VI, a known developmental marker, for collagens and CS-6 for GAGs. In the fourth phase, separations within the ECM can be identified, with notable differences in the spatial distribution of collagen types II and VI. The mature tissue matrix consists predominately of collagen type II, while collagen type VI decreases in proportion to total collagen and presumes a pericellular localization. In addition, the chondroitin sulfation patterns change during neotissue development, as the CS-6 to CS-4 ratio follows a downward trend over time. The composition of this ECM can be directly related to the emergent compressive and tensile mechanical properties of the neotissue.

## References

[1] Hu JC, Athanasiou KA (2003). Structure and Function of Articular Cartilage.. Handbook of Histology Methods for Bone and Cartilage.

[2] Arthritis (2007). Disease Center: Osteoarthritis.. http://www.arthritis.org.

[3] Hu JC, Athanasiou KA (2006). A self-assembling process in articular cartilage tissue engineering.. Tissue Eng.

[4] Elder BD, Athanasiou KA (2008). Effects of confinement on the mechanical properties of self-assembled articular cartilage constructs in the direction orthogonal to the confinement surface.. J Orthop Res.

[5] Hu JC, Athanasiou KA (2006). The effects of intermittent hydrostatic pressure on self-assembled articular cartilage constructs.. Tissue Eng.

[6] Hayes AJ, Hall A, Brown L, Tubo R, Caterson B (2007). Macromolecular organization and in vitro growth characteristics of scaffold-free neocartilage grafts.. J Histochem Cytochem.

[7] Pacifici M, Koyama E, Iwamoto M, Gentili C (2000). Development of articular cartilage: what do we know about it and how may it occur?. Connect Tissue Res.

[8] Morrison EH, Ferguson MW, Bayliss MT, Archer CW (1996). The development of articular cartilage: I. The spatial and temporal patterns of collagen types.. J Anat.

[9] Archer CW, Morrison EH, Bayliss MT, Ferguson MW (1996). The development of articular cartilage: II. The spatial and temporal patterns of glycosaminoglycans and small leucine-rich proteoglycans.. J Anat.

[10] Quarto R, Dozin B, Bonaldo P, Cancedda R, Colombatti A (1993). Type VI collagen expression is upregulated in the early events of chondrocyte differentiation.. Development.

[11] Sherwin AF, Carter DH, Poole CA, Hoyland JA, Ayad S (1999). The distribution of type VI collagen in the developing tissues of the bovine femoral head.. Histochem J.

[12] Lewis S, Crossman M, Flannelly J, Belcher C, Doherty M (1999). Chondroitin sulphation patterns in synovial fluid in osteoarthritis subsets.. Ann Rheum Dis.

[13] Calabro A, Hascall VC, Midura RJ (2000). Adaptation of FACE methodology for microanalysis of total hyaluronan and chondroitin sulfate composition from cartilage.. Glycobiology.

[14] Craig FM, Bentley G, Archer CW (1987). The spatial and temporal pattern of collagens I and II and keratan sulphate in the developing chick metatarsophalangeal joint.. Development.

[15] Kempson GE (1982). Relationship between the tensile properties of articular cartilage from the human knee and age.. Ann Rheum Dis.

[16] Williamson AK, Chen AC, Sah RL (2001). Compressive properties and function-composition relationships of developing bovine articular cartilage.. J Orthop Res.

[17] Williamson AK, Chen AC, Masuda K, Thonar EJ, Sah RL (2003). Tensile mechanical properties of bovine articular cartilage: variations with growth and relationships to collagen network components.. J Orthop Res.

[18] Elder BD, Athanasiou KA (2007). Effects of confinement on the mechanical properties of self-assembled articular cartilage constructs in the direction orthogonal to the confinement surface.. J Orthop Res.

[19] Rosenberg L (1971). Chemical basis for the histological use of safranin O in the study of articular cartilage.. J Bone Joint Surg Am.

[20] Brown AN, Kim BS, Alsberg E, Mooney DJ (2000). Combining chondrocytes and smooth muscle cells to engineer hybrid soft tissue constructs.. Tissue Eng.

[21] Pietila K, Kantomaa T, Pirttiniemi P, Poikela A (1999). Comparison of amounts and properties of collagen and proteoglycans in condylar, costal and nasal cartilages.. Cells Tissues Organs.

[22] Woessner JF (1961). The determination of hydroxyproline in tissue and protein samples containing small proportions of this imino acid.. Arch Biochem Biophys.

[23] Grande-Allen KJ, Calabro A, Gupta V, Wight TN, Hascall VC (2004). Glycosaminoglycans and proteoglycans in normal mitral valve leaflets and chordae: association with regions of tensile and compressive loading.. Glycobiology.

[24] Grande-Allen KJ, Griffin BP, Ratliff NB, Cosgrove DM, Vesely I (2003). Glycosaminoglycan profiles of myxomatous mitral leaflets and chordae parallel the severity of mechanical alterations.. J Am Coll Cardiol.

[25] Athanasiou KA, Agarwal A, Dzida FJ (1994). Comparative study of the intrinsic mechanical properties of the human acetabular and femoral head cartilage.. J Orthop Res.

[26] Mow VC, Kuei SC, Lai WM, Armstrong CG (1980). Biphasic creep and stress relaxation of articular cartilage in compression? Theory and experiments.. J Biomech Eng.

[27] Mow VC, Gibbs MC, Lai WM, Zhu WB, Athanasiou KA (1989). Biphasic indentation of articular cartilage–II. A numerical algorithm and an experimental study.. J Biomech.

[28] Fraser SA, Crawford A, Frazer A, Dickinson S, Hollander AP (2006). Localization of type VI collagen in tissue-engineered cartilage on polymer scaffolds.. Tissue Eng.

[29] Dimicco MA, Kisiday JD, Gong H, Grodzinsky AJ (2007). Structure of pericellular matrix around agarose-embedded chondrocytes.. Osteoarthritis Cartilage.

[30] Poole CA, Ayad S, Schofield JR (1988). Chondrons from articular cartilage: I. Immunolocalization of type VI collagen in the pericellular capsule of isolated canine tibial chondrons.. J Cell Sci.

[31] Poole CA, Ayad S, Gilbert RT (1992). Chondrons from articular cartilage. V. Immunohistochemical evaluation of type VI collagen organisation in isolated chondrons by light, confocal and electron microscopy.. J Cell Sci.

[32] Poole CA (1997). Articular cartilage chondrons: form, function and failure.. J Anat.

[33] Hing WA, Sherwin AF, Poole CA (2002). The influence of the pericellular microenvironment on the chondrocyte response to osmotic challenge.. Osteoarthritis Cartilage.

[34] Haider MA, Schugart RC, Setton LA, Guilak F (2006). A mechano-chemical model for the passive swelling response of an isolated chondron under osmotic loading.. Biomech Model Mechanobiol.

[35] Ruoslahti E, Yamaguchi Y (1991). Proteoglycans as modulators of growth factor activities.. Cell.

[36] Guilak F, Mow VC (2000). The mechanical environment of the chondrocyte: a biphasic finite element model of cell-matrix interactions in articular cartilage.. J Biomech.

[37] Guilak F, Jones WR, Ting-Beall HP, Lee GM (1999). The deformation behavior and mechanical properties of chondrocytes in articular cartilage.. Osteoarthritis Cartilage.

[38] Alexopoulos LG, Haider MA, Vail TP, Guilak F (2003). Alterations in the mechanical properties of the human chondrocyte pericellular matrix with osteoarthritis.. J Biomech Eng.

[39] Alexopoulos LG, Setton LA, Guilak F (2005). The biomechanical role of the chondrocyte pericellular matrix in articular cartilage.. Acta Biomater.

[40] Youn I, Choi JB, Cao L, Setton LA, Guilak F (2006). Zonal variations in the three-dimensional morphology of the chondron measured in situ using confocal microscopy.. Osteoarthritis Cartilage.

[41] Alexopoulos LG, Williams GM, Upton ML, Setton LA, Guilak F (2005). Osteoarthritic changes in the biphasic mechanical properties of the chondrocyte pericellular matrix in articular cartilage.. J Biomech.

[42] Foty RA, Steinberg MS (2005). The differential adhesion hypothesis: a direct evaluation.. Dev Biol.

[43] Oberlender SA, Tuan RS (1994). Spatiotemporal profile of N-cadherin expression in the developing limb mesenchyme.. Cell Adhes Commun.

[44] Oberlender SA, Tuan RS (1994). Expression and functional involvement of N-cadherin in embryonic limb chondrogenesis.. Development.

[45] Bland YS, Ashhurst DE (1996). Development and ageing of the articular cartilage of the rabbit knee joint: distribution of the fibrillar collagens.. Anat Embryol (Berl).

[46] Mow VC, Ratcliffe A, Mow VC, Hayes WC (1997). *Structure and function of articular cartilage and meniscus*,. Basic Orthopaedic Biomechanics..

[47] Platt D, Bird JL, Bayliss MT (1998). Ageing of equine articular cartilage: structure and composition of aggrecan and decorin.. Equine Vet J.

[48] Sorrell JM, Caterson B (1989). Detection of age-related changes in the distributions of keratan sulfates and chondroitin sulfates in developing chick limbs: an immunocytochemical study.. Development.

[49] Bayliss MT, Ali SY (1978). Age-related changes in the composition and structure of human articular-cartilage proteoglycans.. Biochem J.

[50] Athanasiou KA, Rosenwasser MP, Buckwalter JA, Malinin TI, Mow VC (1991). Interspecies comparisons of in situ intrinsic mechanical properties of distal femoral cartilage.. J Orthop Res.

[51] Kawakami Y, Wada N, Nishimatsu SI, Ishikawa T, Noji S (1999). Involvement of Wnt-5a in chondrogenic pattern formation in the chick limb bud.. Dev Growth Differ.

[52] Daumer KM, Tufan AC, Tuan RS (2004). Long-term in vitro analysis of limb cartilage development: involvement of Wnt signaling.. J Cell Biochem.

[53] Oshin AO, Stewart MC (2007). The role of bone morphogenetic proteins in articular cartilage development, homeostasis and repair.. Vet Comp Orthop Traumatol.

[54] Koay EJ, Hoben GM, Athanasiou KA (2007). Tissue engineering with chondrogenically differentiated human embryonic stem cells.. Stem Cells.

[55] Kempson GE, Tuke MA, Dingle JT, Barrett AJ, Horsfield PH (1976). The effects of proteolytic enzymes on the mechanical properties of adult human articular cartilage.. Biochim Biophys Acta.

[56] Asanbaeva A, Masuda K, Thonar EJ, Klisch SM, Sah RL (2007). Regulation of immature cartilage growth by IGF-I, TGF-beta1, BMP-7, and PDGF-AB: role of metabolic balance between fixed charge and collagen network.. Biomech Model Mechanobiol.

[57] Asanbaeva A, Masuda K, Thonar EJ, Klisch SM, Sah RL (2007). Mechanisms of cartilage growth: modulation of balance between proteoglycan and collagen in vitro using chondroitinase ABC.. Arthritis Rheum.

[58] Knight MM, Lee DA, Bader DL (1998). The influence of elaborated pericellular matrix on the deformation of isolated articular chondrocytes cultured in agarose.. Biochim Biophys Acta.

[59] Pullig O, Weseloh G, Swoboda B (1999). Expression of type VI collagen in normal and osteoarthritic human cartilage.. Osteoarthritis Cartilage.

[60] Hashimoto K, Fukuda K, Yamazaki K, Yamamoto N, Matsushita T (2006). Hypoxia-induced hyaluronan synthesis by articular chondrocytes: the role of nitric oxide.. Inflamm Res.

[61] Pavasant P, Shizari T, Underhill CB (1996). Hyaluronan synthesis by epiphysial chondrocytes is regulated by growth hormone, insulin-like growth factor-1, parathyroid hormone and transforming growth factor-beta 1.. Matrix Biol.

[62] Bharadwaj AG, Rector K, Simpson MA (2007). Inducible hyaluronan production reveals differential effects on prostate tumor cell growth and tumor angiogenesis.. J Biol Chem.

[63] Shelton JC, Bader DL, Lee DA (2003). Mechanical conditioning influences the metabolic response of cell-seeded constructs.. Cells Tissues Organs.

[64] Shapiro IM, Leboy PS, Tokuoka T, Forbes E, DeBolt K (1991). Ascorbic acid regulates multiple metabolic activities of cartilage cells.. Am J Clin Nutr.

